# Indium *K*α radiation from a MetalJet X-ray source: the long way to a successful charge-density investigation

**DOI:** 10.1107/S1600576723007203

**Published:** 2023-09-05

**Authors:** Nico Graw, Paul Niklas Ruth, Tobias Ernemann, Regine Herbst-Irmer, Dietmar Stalke

**Affiliations:** a Institut für Anorganische Chemie, Georg-August-Universität Göttingen, Tammannstraße 4, Göttingen, Lower Saxony 37077, Germany; Ecole National Supérieure des Mines, Saint-Etienne, France

**Keywords:** indium radiation, charge density, multipole model, MetalJet, ylide compounds

## Abstract

The MetalJet X-ray source provides indium *K*α radiation with a wavelength even shorter than Ag radiation. This paper reports on problematic spectral impurities and presents possible countermeasures so that collection of data with excellent quality up to a high resolution is possible. It is demonstrated that these data can be used in the refinement of a multipole model, the results of which are used for a topological analysis to assess the bonding situation in a sulfur ylide compound.

## Abstract 2

Enabling of an In *K*α MetalJet source is described, culminating in the first charge-density investigation.

## 1. Introduction

After W. C. Röntgen had discovered X-rays in 1895 (Röntgen, 1895 ▸), his findings were immediately met with great interest in the scientific community and society (Posner, 1970 ▸; Nascimento, 2014 ▸). Accordingly, development of X-ray sources followed quickly. The first patent for an X-ray tube was granted only about four months after Röntgen’s discovery (Nascimento, 2014 ▸). These early Crookes-type tubes were followed by Coolidge tubes using heated metal filaments as electron sources (Coolidge, 1916 ▸, 1925 ▸). In 1927 the beryllium exit window was introduced (Becker, 1927 ▸), and one year later the first rotating anode was presented, which allowed for the use of a higher electron beam power to yield higher X-ray intensities (Gray, 1930 ▸). Since then, commercially available X-ray tubes have been developed for all kinds of purposes (Behling, 2020 ▸), but their key features have not changed. Accordingly, the X-ray flux of home laboratory sources has always been limited by the tolerable heat load of the metal target before melting. This limitation was not overcome until 2003 when the MetalJet X-ray source demonstrated the use of a liquid metal alloy as the anode target (Hemberg *et al.*, 2003 ▸). It was shown that this source can be used to provide gallium (9.3 keV) or indium (24.2 keV) *K*α radiation (Otendal *et al.*, 2008 ▸; Larsson *et al.*, 2011 ▸). However, only the softer gallium radiation has been used for structure determination by single-crystal X-ray diffraction experiments so far (Chan *et al.*, 2018 ▸; Klein *et al.*, 2017 ▸; Liang *et al.*, 2021 ▸; Mehr *et al.*, 2020 ▸; Pan *et al.*, 2017 ▸). Initial experiments showing that diffraction data can also be obtained with indium radiation have been reported recently in a PhD thesis (Nöthling, 2021 ▸).

Herein, we report the collection of single-crystal X-ray diffraction data with the harder indium *K*α radiation from a MetalJet X-ray source, enabling for the first time a standard routine structure solution and refinement. We show that refinement of an independent atom model for data collected on a sulfur ylide crystal is possible. After tackling problems with spectral impurities, for which we present possible remedies, a multipolar refinement and an experimental charge-density analysis within the quantum theory of atoms in molecules (QTAIM; Bader, 1990 ▸) framework are also possible, and the results of these will be discussed in terms of an experimental description of the bonding situation in the investigated sulfur ylide.

## 2. Experimental

### 2.1. Diffraction setup

For our experiments we used a model D2 MetalJet X-ray source from Excillum AB integrated into a Bruker D8 Venture diffractometer with a four-circle goniometer, a Bruker Photon II detector and Montel multilayer optics from Incoatec optimized for indium *K*α radiation. The MetalJet source was run with ExAlloy I1 (68.5% Ga, 21.5% In, 10% Sn) as anode material and operated at 200 W with a 70 kV high-voltage generator. During the course of our work the alloy was exchanged for ExAlloy I3 (75% Ga, 25% In), together with installation of the dynamic adaption technology, allowing the source to be run at 250 W. Lastly, a Dectris Eiger2 CdTe 1M detector was home-implemented for comparison.

## 3. Results

### 3.1. Spectral contamination by Ga *K*α radiation

The multilayer optics used here were optimized for indium *K*α radiation and should monochromatize the X-ray beam. However, the presence of spectral contamination by gallium *K*α radiation was obvious from the first diffraction images, as it gave rise to a separate set of reflections. This is most probably due to total reflection allowing the gallium *K*α radiation (and also bremsstrahlung in the same energy range) to pass the optics. The most straightforward solution to eliminate this spectral impurity is placing an attenuator in the beam path. Since the energy of the gallium radiation is much lower than that of indium, it is in general more strongly attenuated. To devise the ideal thickness of aluminium to be used for sufficient attenuation of the gallium *K*α contamination, but not to decrease unnecessarily the intensity of the indium *K*α radiation, diffraction data were collected with different attenuator thicknesses. One of the well established 2-dimethylsulfuranylidene-l,3-indanedione (YLID; C_11_H_10_O_2_S) crystals (Guzei *et al.*, 2008 ▸) was used for all measurements following identical measurement strategies. Since the *SAINT* (Bruker, 2016 ▸) integration software does not allow for integration with two different wavelengths simultaneously, reflections caused by the gallium *K*α contamination needed to be described by a second unit cell instead. Because the YLID test crystal crystallizes in the orthorhombic space group *P*2_1_2_1_2_1_, an emulated second cell can easily be calculated by multiplication of the original cell parameters with the ratio of wavelengths:

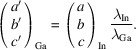

Converting the problem from two different wavelengths to two different cells allows for the data to be treated as a non-merohedral twin

All data sets were integrated with *SAINT* and scaled with *TWINABS* (Sevvana *et al.*, 2019 ▸). After space-group determination with *XPREP* (Sheldrick, 2015*c* ▸) and structure solution with *SHELXT* (Sheldrick, 2015*b* ▸), an identical model was refined for all data sets using *SHELXL* (Sheldrick, 2015*a* ▸) in the graphical user interface *ShelXle* (Hübschle *et al.*, 2011 ▸). The batch scale factor (BASF) obtained from the final refinement was used to assess the relative reduction in gallium *K*α contamination with increasing attenuator thickness. The results are summarized in Table 1 ▸. It is evident that the default attenuator made of 0.75 mm aluminium supplied by Bruker significantly reduces the gallium *K*α contamination but does not suffice to block it beyond a detectable limit. The optimal value for the attenuator was found to be 0.95 mm thickness, as indicated by the lowest values of the BASF and *R* factors. Further increase in attenuator thickness only resulted in a reduction in data and model quality.

### 3.2. Spectral contamination by Sn *K*α radiation

The original alloy used as the anode target material in the MetalJet X-ray source also contained tin, so by design the source also emits Sn *K*α radiation. This was not known to us initially but became apparent after careful inspection of the reflections, which showed tailing towards the beam centre and eventually became split at higher resolutions. This observation is in agreement with the wavelength of Sn *K*α radiation (0.4906 Å) being slightly shorter than that of In *K*α (0.5134 Å) (Deslattes *et al.*, 2005 ▸). Unlike Ga *K*α radiation, Sn *K*α radiation is higher in energy than In *K*α radiation. Therefore, it cannot simply be attenuated as the intensity of In *K*α would decrease even more unless an attenuator were used which exhibits an absorption edge between the In and Sn *K*α energies. With an absorption edge at 24.35 keV, palladium is the only element that fulfils this requirement (Hubbell & Seltzer, 2004 ▸). Hence, data sets with increasing Pd attenuator thickness were collected on an YLID test crystal using identical measurement strategies. Because of the large overlap between reflections from In *K*α and Sn *K*α radiation, a twin refinement was not possible. Instead, the data were integrated with *SAINT* and scaled with *SADABS* (Krause *et al.*, 2015 ▸). Identical models were refined for all data sets using *SHELXL* in the graphical user interface *ShelXle*. Due to the Sn *K*α contribution the reflections were smeared out towards the beam centre. Subsequently, it could be expected that the reduced apparent diffraction angle leads to increased cell volumes. Therefore, the cell volumes of all measurements with palladium and the optimized 0.95 mm Al attenuation were compared with reference data collected using a Bruker D8 Venture four-circle diffractometer with an Incoatec IμS 3.0 Ag source and a Bruker Photon III detector. The results are summarized in Table 2 ▸. As expected, the cell volume decreases slightly with an increase in palladium thickness. However, due to the exponential character of the attenuation effect no clear optimal value for the palladium thickness could be determined. Considering the overall quality of the collected data and the accompanying loss of intensity in In *K*α radiation, we found that 40 µm of palladium offers a good compromise between the remaining intensity and spectral purity that yields sufficiently good data.

**Figure 1:**
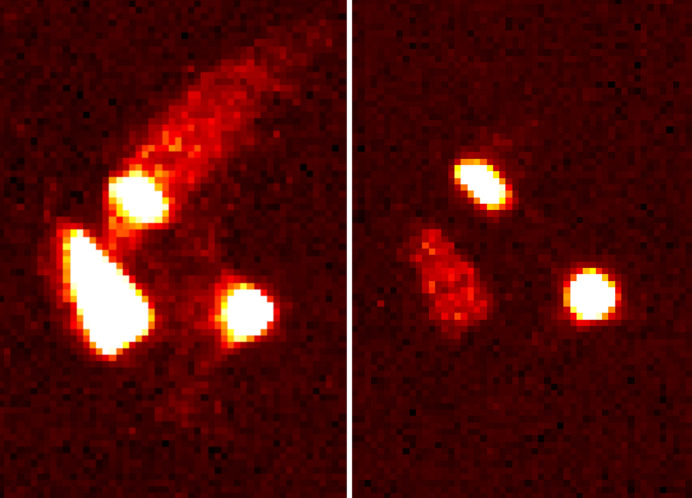
The uncollimated X-ray intensity exiting the Montel optics as detected with the Photon II detector. (Left) With 13.9 mm of aluminium used for attenuation. (Right) With 0.6 mm of palladium used for attenuation.

As a consequence, it became clear that the Sn *K*α contamination has an influence on the alignment of the diffractometer, as was also described by Nöthling (2021 ▸). Fig. 1 ▸ shows the X-ray intensities exiting the Montel optics as recorded on the Photon II detector with either (left) 13.9 mm of aluminium or (right) 0.6 mm of palladium used for attenuation. The relative absorption of the In *K*α radiation in the two cases is about equal. However, the image obtained with palladium attenuation is much cleaner. Palladium clearly aids in suppressing further spectral contamination and, more importantly, gives a well defined doubly diffracted beam. Therefore, we adopted the use of a 600 µm palladium attenuator during the alignment routine to ensure that the alignment was performed with a beam maximized in In *K*α radiation intensity and not in a mixture of In *K*α and Sn *K*α radiation.

### 3.3. Exchange of Montel optics

Since attenuation of any kind always reduces the intensity of the In *K*α radiation as well, we sought a possibility of circumventing the need for attenuation. To check the performance of the X-ray optics we exchanged them for different Montel optics (M2) lent to us by Bruker. These were also manufactured by Incoatec and optimized for In *K*α radiation. For both optics the resulting X-ray beam was characterized with a calibrated pin diode. Measured mean flux densities are given in Table S1 in the supporting information. The peak brilliance of the X-ray beam that could be derived from these measurements was 5.35 × 10^8^ photons (s mm^2^ mrad 0.1% BW)^−1^ for the optics originally installed in the setup (M1) and 3.50 × 10^8^ photons (s mm^2^ mrad 0.1% BW)^−1^ for the M2 optics. Accordingly, the beam shaped by the M1 optics also had a smaller diameter (FWHM) of 44 µm compared with 52 µm for M2. Because of their slightly superior performance also in the refinement results the original optics were kept in the setup.

### 3.4. Exchange of alloy

Since the characteristic tin radiation could not be suppressed by the optics, and palladium attenuation reduces the intensity of In *K*α radiation significantly, discussions with the manufacturers led to a new tin-free alloy being sourced by Excillum AB, which is now available as ExAlloy I3 (65% Ga, 25% In). The whole alloy cycling system was exchanged to avoid contamination from the old alloy. Simultaneously, a dynamic adaption technology was installed, which increases the cathode lifetime and allows for the source to be run at 250 W of power.

## 4. YLID charge-density refinement

Having optimized the diffraction setup to the best of our abilities, we then wanted to collect high-resolution data and perform an experimental charge-density analysis to assess the data quality. For this purpose, we again used the YLID crystal as a benchmark system. Also, the Photon II detector was compared with a Photon III and a Dectris Eiger2 CdTe 1M detector. The Eiger2 CdTe detector yielded the best data when an energy cut-off at half the In *K*α energy was used to give effective discrimination between the intensities caused by gallium *K*α radiation. All results discussed in this paper stem from that detector. Details of the comparison and of the data processing are discussed in the accompanying paper (Ruth *et al.*, 2023 ▸). A multipole refinement according to the Hansen–Coppens multipole formalism (Hansen & Coppens, 1978 ▸) employing all data with non-negative intensities was performed on *F* ^2^ using the refinement program *XDLSM* implemented in *XD2016* (Volkov *et al.*, 2016 ▸). The results are summarized and compared with an independent-atom model (IAM) refinement of the same data in Table 3 ▸. Normal probability (Abrahams & Keve, 1971 ▸), DRK (Stash, 2007 ▸; Zavodnik *et al.*, 1999 ▸; Zhurov *et al.*, 2008 ▸) and Henn–Meindl (Meindl & Henn, 2008 ▸) plots together with residual and deformation density maps are shown in Figs. S1–S4. Information on the multipole model and refinement strategy is given in Tables S2 and S3 in the supporting information.

**Figure 2:**
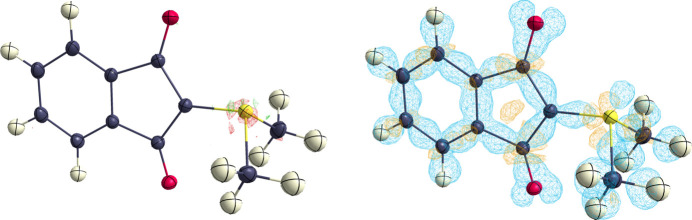
(Left) A residual density map with isosurfaces at ±0.05 e Å^−^^3^. Positive contours are plotted with green lines and negative contours with red lines. (Right) A deformation density map with isosurfaces at ±0.09 e Å^−^^3^. Positive contours are plotted with blue lines and negative contours with orange lines. Graphics were created using *MoleCoolQt* (Hübschle & Dittrich, 2011 ▸).

Fig. 2 ▸ shows 3D maps of the residual and deformation densities. At the isosurface level of ±0.05 e Å^−3^ almost no residual density is left, indicating that the multipole model describes the experimentally determined charge-density distribution excellently. The remaining residuals are located around the sulfur atom, which is the heaviest atom in the structure. The deformation density map shows electron density allocated mostly along chemical bonds, as one would expect, and additionally indicates accumulation of electron density in the non-bonding regions around the sulfur atom and both oxygen atoms, not described by the IAM refinement.

**Figure 3:**
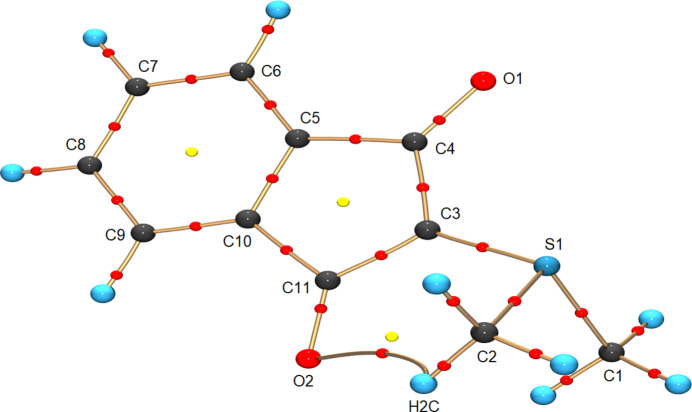
A molecular graph of YLID. Red and yellow dots represent bond and ring critical points, respectively.

The experimental charge-density distribution of the YLID structure was then analysed according to QTAIM (Bader, 1990 ▸). The molecular graph is shown in Fig. 3 ▸. In addition to the expected features, a bond path and a bond critical point (BCP) between atoms O2 and H2*C* were found, resulting in an additional ring critical point. The bond path between O2 and H2*C* is highly curved, as one would expect for a weak intramolecular hydrogen bond (Row, 2020 ▸; Munshi & Guru Row, 2005 ▸; Krawczuk & Gryl, 2018 ▸), whereas all other bond paths show little to no curvature, indicating strong covalent bonding.

Values of selected properties at the BCPs for chosen bonds are given in Table 4 ▸ (full data in Table S5). These confirm the general description of bonding that has already emerged from the molecular graph. With high electron density and negative values of the Laplacian at the BCP, as well as a ratio of |*V*_BCP_|/*G*_BCP_ > 2, most bonds can be characterized as shared shell interactions (Bianchi *et al.*, 2000 ▸). The only exception is the interaction between O2 and H2*C* which also, in accordance with the values listed in Table 4 ▸, can be classified as a hydrogen bond of moderate strength.

**Figure 4:**
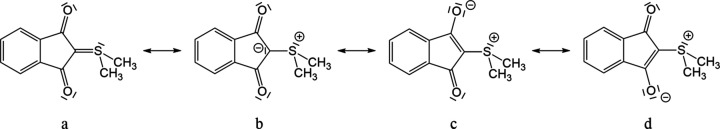
(*a*) Ylene and (*b*) ylide resonance structures of 2-di­methyl­sufuran­ylidene-l,3-indanedione (YLID), with (*c*) and (*d*) additional charge delocalization across the carbonyl groups.

Since the investigated compound belongs to the class of sulfur ylides, it is of particular interest to investigate the bonds involving the sulfur atom. As shown in Fig. 4 ▸, the compound can be described by ylenic and ylidic resonance structures. The former [Fig. 4 ▸(*a*)] violates the octet rule by introduction of hypervalency, although this has been shown to be unreasonable for sulfur compounds (Kutzelnigg, 1984 ▸; Reed & von Ragué Schleyer, 1990 ▸; Schmøkel *et al.*, 2012 ▸; Stalke, 2016 ▸, 2021 ▸). The ylide resonance structure [Fig. 4 ▸(*b*)], on the other hand, suggests pyramidalization of the involved carbon atom. This is not obvious from the geometry of the compound, since the sum of the angles around C3 is 359.73 (2)° and S1 is dislocated from the plane of the indanedione group by just 0.265 Å. However, this might be explained by delocalization of the electron density associated with the lone pair across the two carbonyl groups [Figs. 4 ▸(*c*) and 4 ▸(*d*)].

The results of the topological analysis can be used to assess the bonding situation experimentally. The S1—C3 bond path (1.7122 Å) is significantly shorter than S1—C1 and S1—C2 (mean value 1.7936 Å). This should not be mistaken as a hint to the sulfur ylenic character [Fig. 4 ▸(*a*)] in this class of compounds (Gololobov *et al.*, 1987 ▸). The bond is shorter than those to the methyl groups because an *sp* ^2^ carbon atom is simply smaller in radius than an *sp* ^3^. All S—C bonds fit almost perfectly to other sulfur ylide bonds, even from the charge-density perspective (Leusser *et al.*, 2004 ▸; Deuerlein *et al.*, 2008 ▸; Walfort & Stalke, 2001 ▸). They are best described as mostly covalent single bonds. However, the ratio of |*V*_BCP_|/*G*_BCP_ is slightly smaller for S1—C3 (2.40) than for S1—C1 and S1—C2 (mean value 2.53), indicating a higher ionic contribution to the bonding in the first case.

Comparison with the properties of *e.g.* O1—C4 (*R*_(*A*—*B*)_ = 1.2327 Å, ρ_BCP_ = 2.80 e Å^−3^, ∇^2^ρ_BCP_ = −29.40 e Å^−5^) or C5—C6 (*R*_(*A*—*B*)_ = 1.3842 Å, ρ_BCP_ = 2.17 e Å^−3^, ∇^2^ρ_BCP_ = −19.53 e Å^−5^), for which significant double-bond character can be expected, makes it even clearer that description of S1—C3 (ρ_BCP_ = 1.48 e Å^−3^, ∇^2^ρ_BCP_ = −6.90 e Å^−5^) as a double bond is not valid.

Delocalization of electron density along the bonds C3—C4—O1 and C3—C11—O2, as indicated in Figs. 4 ▸(*c*) and 4 ▸(*d*), is also evident from the obtained properties. The bond paths of C3—C4 (1.4429 Å) and C3—C11 (1.4365 Å) are shorter than those for C4—C5 (1.5063 Å) and C10—C11 (1.5019 Å). Additionally, the values of the electron density and the Laplacian at the BCPs between C3—C4 (1.92 e Å^−3^ and −13.41 e Å^−5^, respectively) and C3—C11 (1.95 e Å^−3^ and −14.95 e Å^−5^, respectively) agree with a partial double-bond character when compared with those at the BCPs between C4—C5 (1.77 e Å^−3^ and −12.51 e Å^−5^, respectively) and C10—C11 (1.78 e Å^−3^ and −12.75 e Å^−5^, respectively). This is also supported by the obtained ellipticities of 0.28 and 0.24, showing the clear deviation from cylindrical symmetry of the electron density along C3—C4 and C3—C11, respectively, while those along C4—O1 (0.09) and C11—O2 (0.09) are strongly reduced.

To see if the charge-separated ylide description of the bonding can be further consolidated, integrated QTAIM charges were investigated (full data in Table S6). Atom C3 is more negatively charged (−0.21 e) than C1 (−0.16 e) and C2 (−0.15 e), whereas S1 bears a positive charge (0.28 e). In accordance with the resonance structures in Figs. 4 ▸(*c*) and 4 ▸(*d*), significant negative QTAIM charges are also found for O1 (−1.01 e) and O2 (−1.00 e), as well as positive charges on C4 (0.79 e) and C11 (0.77 e), indicative of a resonance structure with both O atoms negatively charged and the positive charge on either C4 or C11.

**Figure 5:**
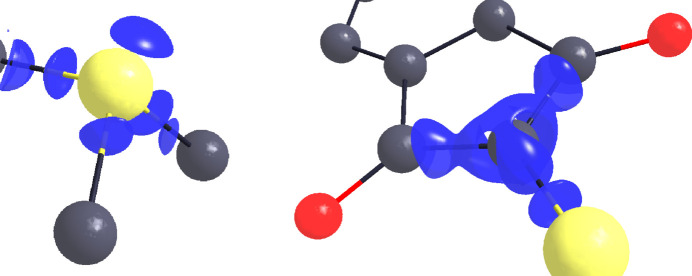
Calculated Laplacians of the experimental electron density in a 2 × 2 × 2 Å cube around (left) S1 and (right) C3, with isosurfaces at 0.4 e Å^−5^.

The three-dimensional distribution of the Laplacian was inspected to see if valence-shell charge concentrations (VSCCs) related to the lone pair of electrons shown in the ylide resonance structure [Fig. 4 ▸(*a*)] could be found. Visualizations of the Laplacian are shown in Fig. 5 ▸. In the case of S1, three VSCCs can be found along the bonds pointing towards the respective bonding partner. An additional cleanly separated VSCC correlated to the expected lone pair is found in the non-bonding region, which is oriented away from the three S—C bonds. For C3, on the other hand, no separated VSCC indicating a lone pair of electrons can be observed. This is reminiscent of the picolyl carbanion, where the charge is also delocalized in the aromatic ring (Ott *et al.*, 2009 ▸; Macchi, 2009 ▸).

Taking all of this into account, the bonding situation in the investigated compound is best described by a zwitterionic ylide rather than an ylene structure. The suggested lone pair at C3 from the Lewis diagram in Fig. 4 ▸(*b*) might disagree with its low QTAIM charge and the overall geometry, but this can be rationalized by delocalization of the electron density from C3 via the five-membered ring to both neighbouring carbonyl groups.

## 5. Conclusions

We have shown that even high-quality Montel multilayer optics optimized for In *K*α radiation are not capable of purifying radiation emitted from a MetalJet X-ray source run with either ExAlloy I1 or ExAlloy I3. While the influence of the Ga *K*α yields additional spots in the diffraction pattern and as such can be characterized by a pseudo-twin refinement, the influence of Sn *K*α in Exalloy I1 is subtler and can be seen as a confounding factor in the determination of unit cells. The attenuator material used for aligning the diffraction setup needs to be adapted to the employed alloy, where palladium is crucial if one employs ExAlloy I1 for use with In *K*α. In any case, additional attenuation with aluminium or palladium is necessary, indeed crucial, during alignment of the diffraction setup.

We have demonstrated the ability of this setup to collect high-resolution X-ray diffraction data of excellent quality on a crystal of 2-di­methyl­sufuran­ylidene-l,3-indanedione and performed a topological analysis of the experimental electron density, showing that the compound is best described with a charge-separated ylide structure rather than an ylene resonance structure.

In addition to being an interesting result in itself, this investigation has also demonstrated the potential of the MetalJet source for application in charge-density determination and subsequent analysis of the obtained density within the QTAIM framework.

## Supplementary Material

Crystal structure: contains datablock(s) I. DOI: 10.1107/S1600576723007203/nb5353sup1.cif

Structure factors: contains datablock(s) Ylid_MM_In_Eiger. DOI: 10.1107/S1600576723007203/nb5353Isup2.hkl

Additional tables and figures. DOI: 10.1107/S1600576723007203/nb5353sup3.pdf

CCDC reference: 2288995

## Figures and Tables

**Table 1:** Results from twin refinements For *R*_int_, the integration was done with one orientation matrix.

Al thickness (mm)	*R*_int_ (0.48 Å)	*R*1 [*I* < 2 σ(*I*)] (0.48 Å)	BASF (%)
–	4.78	8.88	62.8 (9)
0.75	4.30	3.99	0.55 (4)
0.85	4.09	3.76	0.28 (4)
0.95	3.86	3.62	0.12 (3)
1.25	4.31	3.84	0.11 (3)

**Table 2:** Differences in cell volume *V*, quality indicators and average intensity 〈*I*〉 obtained with different attenuators For reference, the unit-cell volume determined using Ag *K*α was 958.1 (4) Å^3^. All data were collected up to a resolution of 0.45 Å.

	Attenuator					
Parameter	950 µm Al	25 µm Pd	40 µm Pd	50 µm Pd	75 µm Pd	100 µm Pd
*V* (Å^3^)	963.3 (4)	958.3 (4)	957.8 (4)	957.8 (4)	957.4 (4)	957.3 (4)
*R*1 [*I* < 2 σ(*I*)]	3.72%	3.41%	3.24%	3.33%	3.62%	3.84%
*wR*2 (all data)	9.38%	8.53%	8.25%	8.34%	8.76%	9.42%
ρ_min_/ρ_max_ (e Å^−3^)	−0.28/0.54	−0.36/0.48	−0.28/0.46	−0.31/0.45	−0.41/0.43	−0.43/0.49
〈*I*〉	713.31	1045.69	740.97	675.62	515.20	398.59

**Table:** *e*_gross_ was calculated as published by Meindl & Henn (2008 ▸). GOF is goodness of fit.

IAM refinement	
Data/restraints/parameters	11038/0/129
*R*1/*wR*2 (*I* > 2σ)	2.01%/6.33%
*R*1/*wR*2 (all data)	2.11%/6.39%
ρ_min_/ρ_max_ (e Å^−3^)	−0.190/0.466
*e*_gross_ (e)	17.6

**Table:** 

MM refinement	
Data/parameters	11000/261
*R*1(*F* ^2^)/*wR*1(*F* ^2^) (all data)	0.99%/1.40%
GOF	1.1496
ρ_min_/ρ_max_ (e Å^−3^)	−0.13/0.08
*e*_gross_ (e)	5.63
Maximum shift/s.u. (Å)	0.52 × 10^−6^

**Table 4:** Selected properties at the BCP derived from experimentally determined electron density for YLID *R* is the bond path, ρ_BCP_ the electron density at the BCP, ∇^2^ρ_BCP_ the Laplacian at the BCP, ɛ the ellipticity, *G*_BCP_ the kinetic energy density at the BCP and *V*_BCP_ the potential energy density at the BCP.

Bond (*A*—*B*)	*R*_(*A*—*B*)_ (Å)	*R*_(*A*—BCP)_ (Å)	*R*_(*B*-BCP)_ (Å)	ρ_BCP_ (e Å^−3^)	∇^2^ρ_BCP_ (e Å^−5^)	ɛ	|*V*_BCP_|/*G*_BCP_
S1—C1	1.7985	0.9575	0.8409	1.31	−6.91	0.06	2.51
S1—C2	1.7886	0.9528	0.8358	1.33	−7.23	0.05	2.54
S1—C3	1.7122	0.9062	0.8060	1.48	−6.90	0.10	2.40
C3—C4	1.4429	0.6861	0.7568	1.92	−13.41	0.28	2.54
C3—C11	1.4365	0.6784	0.7581	1.95	−14.95	0.24	2.60
C4—C5	1.5063	0.7550	0.7513	1.77	−12.51	0.08	2.59
C5—C6	1.3842	0.6852	0.6690	2.17	−19.53	0.21	2.68
C10—C11	1.5019	0.7492	0.7527	1.78	−12.75	0.08	2.60
O1—C4	1.2327	0.7899	0.4428	2.80	−29.40	0.09	2.67
O2—C11	1.2375	0.7899	0.4476	2.79	−30.09	0.09	2.70
O2⋯H2*C*	2.5160	1.4360	1.0799	0.08	1.07	1.29	0.83
