# Supplementary material for: Indium Kα radiation from a MetalJet X-ray source: the long way to a successful charge-density investigation
Source: J Appl Crystallogr. 2023 Sep 5;56(Pt 5):1315–21. doi: 10.1107/S1600576723007203 (PMC10543667; doi:10.1107/S1600576723007203)
Supplement: Supplementary file 3 [file j-56-01315-sup3.pdf]

JOURNAL OF APPLIED CRYSTALLOGRAPHY

Volume 56 (2023)

Supporting information for article:

Indium *K*α radiation from a MetalJet X-ray source: the long way to a successful charge-density investigation

Nico Graw, Paul Niklas Ruth, Tobias Ernemann, Regine Herbst-Irmer and Dietmar Stalke

Table S1 Experimental data for the comparison of Montel optics performance.

**Pinhole diameter [mm]**	**Optics M1 mean flux density [cps/mm²]**	**Optics M2 mean flux density [cps/mm²]**
1.128	$1.60{\cdot}10^7$	$1.41{\cdot}10^7$
0.739	$3.66{\cdot}10^7$	$3.24{\cdot}10^7$
0.506	$7.53{\cdot}10^7$	$6.69{\cdot}10^7$
0.293	$2.11{\cdot}10^8$	$1.89{\cdot}10^8$
0.200	$4.17{\cdot}10^8$	$3.70{\cdot}10^8$
0.149	$6.67{\cdot}10^8$	$5.57{\cdot}10^8$
0.098	$1.18{\cdot}10^9$	$9.74{\cdot}10^8$
0.077	$1.50{\cdot}10^9$	$1.21{\cdot}10^9$
0.048	$2.56{\cdot}10^9$	$1.87{\cdot}10^9$
0.028	$3.49{\cdot}10^9$	$2.39{\cdot}10^9$

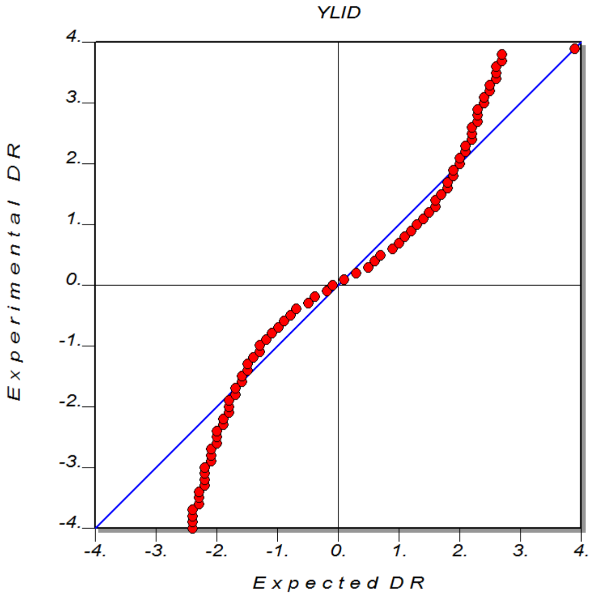

Figure S1 Normal probability plot.

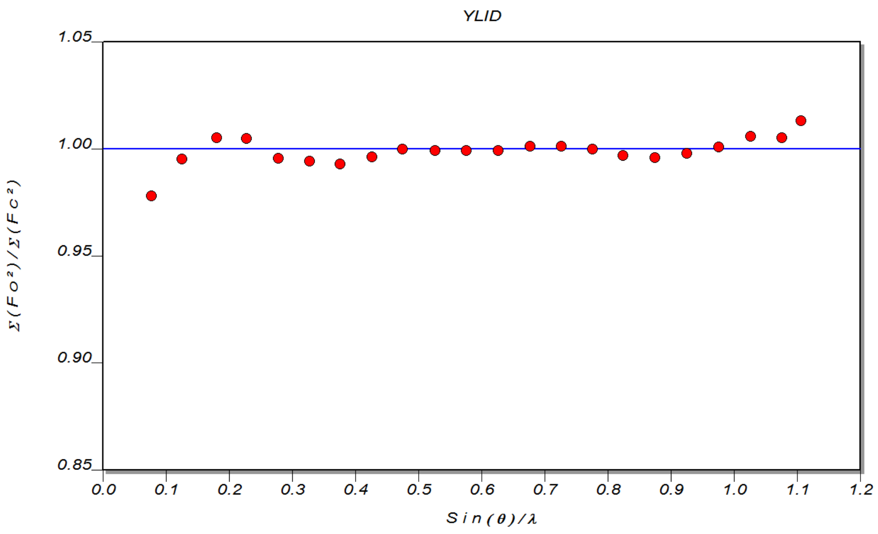

Figure S2 DRK plot.

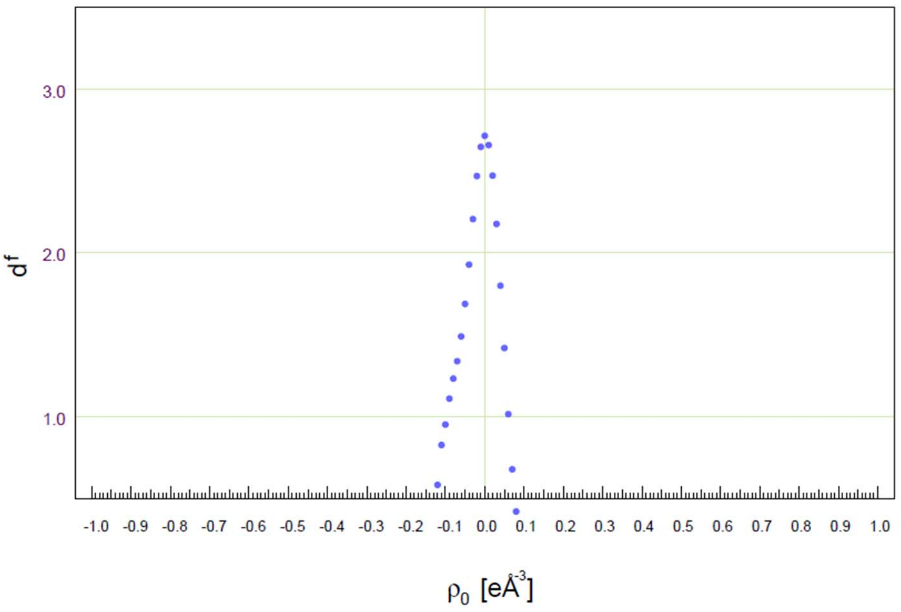

Figure S3 Henn-Meindl plot.

Table S2 Local coordinate system.

ATOM	ATOM0	AX1	ATOM1	ATOM2	AX2	KAP	SITESYM	CHEMCON
S1	C3	Z	S1	DUM0	Y	1	mX	
O1	C4	Z	O1	C3	Y	2	mXmY2Z	
O2	C11	Z	O2	C3	Y	2	mXmY2Z	O1
C1	S1	Z	C1	H1A	Y	3	mX	
C2	S1	Z	C2	H2A	Y	3	mX	C1
C3	S1	Z	C3	C4	Y	4	mX	
C4	O1	Z	C4	C3	Y	4	mX	
C5	C6	Z	C5	C4	Y	4	mX	
C6	C7	Z	C6	C5	Y	4	mX	
C7	C6	Z	C7	C8	Y	4	mX	
C8	C9	Z	C8	C7	Y	4	mX	C7
C9	C8	Z	C9	C10	Y	4	mX	C6
C10	C9	Z	C10	C11	Y	4	mX	C5
C11	O2	Z	C11	C3	Y	4	mX	C4
H1A	C1	Z	H1A	H1B	Y	5	cyl	
H1B	C1	Z	H1B	H1C	Y	5	cyl	H1A
H1C	C1	Z	H1C	H1B	Y	5	cyl	H1A
H2A	C2	Z	H2A	H2C	Y	5	cyl	H1A
H2B	C2	Z	H2B	H2C	Y	5	cyl	H1A
H2C	C2	Z	H2C	H2A	Y	5	cyl	H1A
H6	C6	Z	H6	C5	Y	5	cyl	
H7	C7	Z	H7	C8	Y	5	cyl	H6
H8	C8	Z	H8	C7	Y	5	cyl	H6
H9	C9	Z	H9	C10	Y	5	cyl	H6
DUM0	0.26073 0.78226 0.22343							

Table S3 Refinement strategy. Abbreviations: Sf: scale factor; MP: multipoles; M: monopoles; D: dipoles; Q: quadrupoles; O: octupoles; H: hexadecapoles, U: Uij, k: kappa, k': kappa prime, GOF. Goodness of Fit. Every new added parameter is marked in red.

Step	Refined parameters	Data	MP param.	Param.	Data/parameter ratio	GOF	$R1(F^2)$
1	Sf	9696	0	1	9696	6.1978	3.58 %
2	Sf, M	9696	9	10	969.6	5.7379	3.49 %
3	Sf, MDQOH	9696	93	94	103.1	2.2261	1.68 %
4	Sf, MDQOH, U	9696	93	178	54.5	2.0145	1.54 %
5	Sf, MDQOH, U, XYZ	9696	93	220	44.1	1.7667	1.36 %
6	Sf, MDQOH, U, XYZ, k	9696	97	224	43.3	1.6958	1.26 %
7	Sf, H-XYZ	1001	0	31	32.3	3.0355	1.06 %
8	Sf, MDQOH, U, XYZ, k	9696	97	224	43.3	1.3825	1.10 %
9	Sf, k'	9696	4	5	1939.2	1.3557	1.09 %
10	Sf, MDQOH, U, XYZ, k	9696	97	224	43.3	1.3622	1.08 %
11	Sf, MDQOH, U, XYZ, k, sigobs 0	11000	97	224	49.1	1.2835	1.09 %
12	Sf, MDQOH, DQOH, U, XYZ, k, sigobs 0, O: cyl → mXmY2Z	11000	101	228	48.2	1.1896	1.03 %
13	Sf, MDQOH, DQOH, U, XYZ, k, sigobs 0, C1, C3, C4: 3ZmX → mXmY2Z	11000	121	248	44.4	1.1665	1.02 %
14	Sf, MDQOH, U, XYZ, k, sigobs 0, H: Q	11000	123	250	44.0	1.1496	0.99 %

Table S4 Differences in the mean-square displacement amplitudes (DMSDA) (1×10^{-4} Å^2) along atomic vectors.

ATOM	ATOM	DIST	DMSDA	ATOM	ATOM	DIST	DMSDA
S1	C1	1.7984	8	C5	C6	1.3842	5
S1	C2	1.7885	6	C5	C10	1.3988	−1
S1	C3	1.7121	−2	C6	C7	1.4036	0
O1	C4	1.2327	1	C7	C8	1.3969	−2
O2	C11	1.2375	2	C8	C9	1.4014	0
C3	C4	1.4423	−1	C9	C10	1.3837	−5
C3	C11	1.4357	−3	C10	C11	1.5016	4
C4	C5	1.5060	−3				

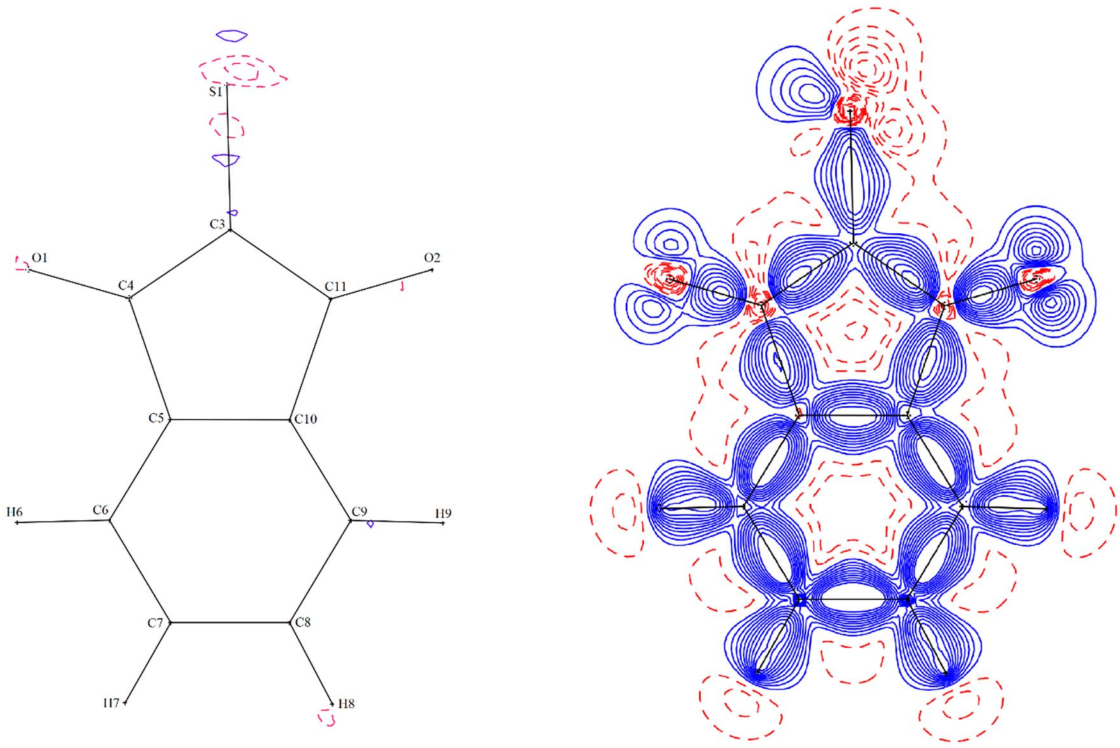

Figure S4 Left: Residual density map. Right: Deformation density map. Contours in both cases are shown at ± {0.05 0.10 0.15 0.20 0.25 0.30 0.35 0.40 0.45 0.50 0.55} eÅ^{-3} with positive values in blue and negative values in red.

Table S5 Calculated BCPs and local energy density properties for YLID. R: bond path; ρ_{BCP} electron density at BCP; $\nabla^2\rho_{BCP}$: Laplacian at BCP; ε ellipticity; G_{BCP}: kinetic energy density at BCP; V_{BCP}: potential energy density at BCP; H_{BCP}: total energy density at BCP.

A	B	$R_{(A-B)}$ [Å]	$R_{(A-BCP)}$ [Å]	$R_{(B-BCP)}$ [Å]	ρ_{BCP} [eÅ^{-3}]	$\nabla^2\rho_{BCP}$ [eÅ^{-5}]	ε	G_{BCP} [E_hÅ^{-3}]	V_{BCP} [E_hÅ^{-3}]	H_{BCP} [E_hÅ^{-3}]
S1	C1	1.7985	0.9575	0.8409	1.31	−6.91	0.06	0.94	−2.36	−1.42
S1	C2	1.7886	0.9528	0.8358	1.33	−7.23	0.05	0.95	−2.41	−1.46
S1	C3	1.7122	0.9062	0.8060	1.48	−6.90	0.10	1.22	−2.93	−1.70
O1	C4	1.2327	0.7899	0.4428	2.80	−29.40	0.09	3.10	−8.27	−5.16
O2	C11	1.2375	0.7899	0.4476	2.79	−30.09	0.09	3.03	−8.17	−5.14
O2	H2C	2.5160	1.4360	1.0799	0.08	1.07	1.29	0.06	−0.05	0.01
C1	H1A	1.0772	0.7033	0.3739	1.79	−20.58	0.03	1.17	−3.77	−2.61
C1	H1B	1.0771	0.7046	0.3725	1.80	−20.91	0.05	1.18	−3.81	−2.64
C1	H1C	1.0772	0.7050	0.3722	1.80	−20.84	0.05	1.18	−3.81	−2.64
C2	H2A	1.0771	0.7032	0.3739	1.79	−20.62	0.03	1.17	−3.78	−2.61
C2	H2B	1.0771	0.7052	0.3720	1.81	−20.86	0.05	1.18	−3.82	−2.64
C2	H2C	1.0772	0.7047	0.3724	1.80	−20.86	0.05	1.18	−3.81	−2.64
C3	C4	1.4429	0.6861	0.7568	1.92	−13.41	0.28	1.75	−4.45	−2.69
C3	C11	1.4365	0.6784	0.7581	1.95	−14.95	0.24	1.74	−4.53	−2.79
C4	C5	1.5063	0.7550	0.7513	1.77	−12.51	0.08	1.49	−3.86	−2.37
C5	C6	1.3842	0.6852	0.6690	2.17	−19.53	0.21	2.02	−5.42	−3.39
C5	C10	1.3990	0.6995	0.6995	2.12	−17.78	0.18	1.98	−5.20	−3.22
C6	C7	1.4036	0.6745	0.7291	2.08	−17.52	0.18	1.90	−5.02	−3.12
C6	H6	1.0836	0.7447	0.3389	1.76	−17.03	0.05	1.27	−3.73	−2.46
C7	C8	1.3973	0.6985	0.6987	2.14	−18.72	0.21	1.99	−5.29	−3.30
C7	H7	1.0831	0.7406	0.3425	1.75	−16.83	0.04	1,26	−3.69	−2.43
C8	C9	1.4015	0.7280	0.6734	2.08	−17.68	0.18	1.90	−5.05	−3.14
C8	H8	1.0832	0.7408	0.3424	1.75	−16.72	0.04	1.26	−3.68	−2.43
C9	C10	1.3838	0.6988	0.6850	2.18	−19.55	0.21	2.03	−5.42	−3.39
C9	H9	1.0836	0.7447	0.3389	1.76	−17.01	0.05	1.27	−3.73	−2.46
C10	C11	1.5019	0.7492	0.7527	1.78	−12.75	0.08	1.50	−3.90	−2.40

Table S6 QTAIM charges.

Atom	charge [e]	Atom	charge [e]
S1	0.28	C10	−0.08
O1	−1.01	C11	0.77
O2	−1.00	H1A	0.10
C1	−0.16	H1B	0.10
C2	−0.15	H1C	0.10
C3	−0.21	H2A	0.10
C4	0.79	H2B	0.10
C5	−0.07	H2C	0.10
C6	−0.08	H6	0.18
C7	−0.15	H7	0.17
C8	−0.15	H8	0.17
C9	−0.07	H9	0.18
